# Normative Data for Nonstrabismic Binocular Vision Parameters in African Schoolchildren

**DOI:** 10.1097/OPX.0000000000001706

**Published:** 2021-06-03

**Authors:** Charles Darko-Takyi, Vanessa R. Moodley, Samuel B. Boadi-Kusi

**Affiliations:** 1 Discipline of Optometry, University of KwaZulu-Natal, Durban, South Africa; 2 Department of Optometry and Vision Science, University of Cape Coast, Cape Coast, Ghana; ∗ cdarko-takyi@ucc.edu.gh;charles.darko-takyi@ucc.edu.gh

## Abstract

With reported population differences in parameters of nonstrabismic binocular vision, the present study investigated and reports normative data among a sample of African children.

**PURPOSE:**

This study aimed to determine expected binocular visual function data among schoolchildren in the Central Region of Ghana.

**METHODS:**

The study used a prospective cross-sectional design and used a multistage cluster sampling approach. Eligible normal participants selected through the administration of Convergence Insufficiency Symptom Survey questionnaire (score <16) and preliminary vision screening underwent comprehensive binocular vision testing. Only data for participants who expressed no difficulty with the specific procedures were analyzed.

**RESULTS:**

A total of 1261 normal participants (11 to 17 years [mean, 14.75 ± 1.530 years]) were selected for comprehensive binocular vision testing in the normative data study. The means with ±1 standard deviation for normative data for the nonstrabismic binocular vision parameters include the following: accommodative target near point of convergence (NPC) break (6.10 ± 1.67 cm), NPC recovery (8.17 ± 1.67 cm), fixation light with red-green anaglyph (RG NPC) break (8.51 ± 2.43 cm), RG NPC recovery (10.95 ± 2.60 cm), cover test distance phoria (0.12 ± 0.79 exophoria), cover test near phoria (2.1 ± 2.3 exophoria), modified Thorington test near phoria (1.9 ± 2.5 exophoria), negative relative accommodation (+2.54 ± 0.75 D), positive relative accommodation (−2.58 ± 0.81 D), and the accommodative convergence over accommodation ratio (2.80 ± 1.07:1). Age (in years)-predicted normal linear regression equations for NPC break (5.13 + 0.07 × age), RG NPC break (10.00 − 0.10 × age), RG NPC recovery (12.83 − 0.13 × age), positive relative accommodation (2.05 + 0.04 × age), and the gradient accommodative convergence over accommodation ratio (3.97 − 0.08 × age) serve as a guide.

**CONCLUSIONS:**

The study provides expected data that optometrists may use with similar aged Black African populations.

## Abstract 2

Supplemental digital content is available in the text.

**Figure:**
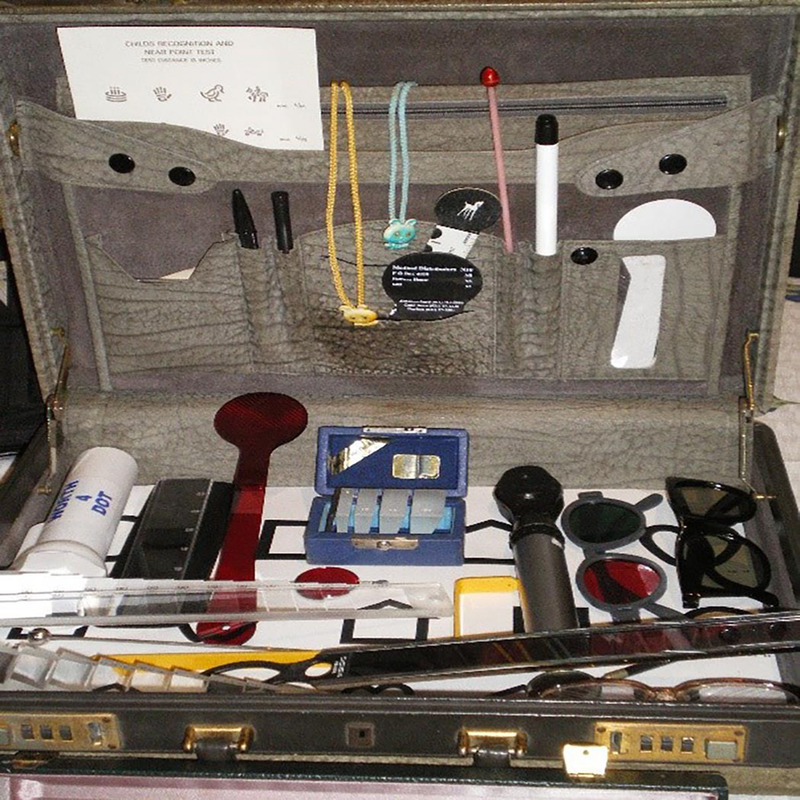


## 

Different normative values for parameters of nonstrabismic binocular vision exist in the literature and have been used worldwide as guidelines to diagnose and treat binocular vision anomalies. Notable among them are Morgan's table of expected findings,^1^ a modified Morgan's table,^2^ Optometric Extension Program table of expected findings,^3,4^ Saladin and Sheedy normative values,^5^ and Scheiman and Wick's table of expected values for binocular vision testing, which is generally considered as the clinical standard.^3^ Reference guidelines for these values date back to old studies mainly conducted on White American populations,^6^ making the application of these normative values when analyzing binocular vision case results in other ethnic populations challenging.^6–8^ Clinicians in the African continent conventionally made the diagnosis and management of nonstrabismic binocular vision disorders by comparing their clinic test results with these established standard normative values.^6–8^ This practice, however, may be inappropriate because the literature acknowledges population differences in normative data for visual function parameters of nonstrabismic binocular vision.^9–11^ Variables such as race, ethnicity, and age define a population and are known to influence refractive status^7,12^ and in turn binocular vision^13–15^ because of the differences in ocular anatomy.^16,17^ There is therefore a need for population-specific normative data for parameters of nonstrabismic binocular vision.

All known binocular vision case analysis techniques require clinic test results to be compared with established normative data.^3^ This is a pre-requisite to classifying patients as having normal or abnormal binocular vision status.^6,9^ Recently, studies have sought to define cutoff normative clinical values for specific tests and thus introduced different values among different populations.^7,9,11,14,18,19^ According to Hussaindeen et al.,^9^ “to optimize the sensitivity and specificity of diagnosis, ethnicity-specific cut-off values for binocular vision parameters are mandatory.”

In Africa, there is a single study among South African children^11^ that reported normative data for visual function parameters for nonstrabismic binocular vision. If one considers the evidence of ethnic variability, these data are technically applicable to 14- to 17-year-old Black South African children only. In the absence of additional African studies to determine normative data, the applicability of the parameters found to other African children remains unknown. Access to education is growing in Africa, and children are exposed to an exponential increase in near-point devices with their associated demands on the binocular vision system. To ensure accurate and reliable diagnosis and management of nonstrabismic binocular vision disorders in African children, there is the need for standard population-specific normative data for the different parameters of nonstrabismic binocular vision disorders. This study was designed to sample normal junior high school children in the central region of Ghana to determine normative data for visual function parameters of nonstrabismic binocular vision. It will also provide an opportunity to compare not only with the White population studies but also with the nonstrabismic binocular vision findings of another African sample.

## METHODS

### Ethical Consideration

This population-based, prospective, cross-sectional study conformed to the tenets of the Declaration of Helsinki and was ethically approved by the Biomedical Research Ethics Committee of the University of KwaZulu Natal and Ghana Health Service Ethics Review Committee. Parents and guardians of participants gave written informed consent, and participants gave written assent after the authors explained the study to them. Each participant was educated that he/she could opt out of the study at any stage if he/she wished to do so.

### Participant Selection

The study used a multistage, stratified, cluster sampling technique to select participants. The 20 districts within the central region of Ghana were clustered into five based on their proximity. The various localities within the districts in each cluster were stratified into two, namely, rural and urban areas based on the Ghana 2010 Population and Housing Census definition. Within each of the strata, one or two schools were randomly selected from each cluster. Children in the first selected school were randomly selected and examined, and in cases where their numbers were not up to that required for the study, children in the second selected school were recruited into the study.

Estimated normative minimum sample sizes were calculated for each of the nonstrabismic binocular vision parameters (Appendix Table A1, available at http://links.lww.com/OPX/A495) using the formula *n* = [*Z*_1 − α/2_^2^SD^2^]/*d*^2^, where *Z*_1 − α/2_ represented standard normal variate at 95% confidence interval (*P* < .05) = 1.96; SD represented the standard deviation of current standards,^3^ and *d* represented the absolute allowable error in estimating values. As indicated (Appendix Table A1, available at http://links.lww.com/OPX/A495), the minimum sample size of 984 was ideal to determine normative data for the parameter with the highest standard deviation to achieve acceptable precision. With an expected minimum sample size of 197 for each cluster, several (not less than 99) normal participants were expected to be randomly selected from each rural and each urban stratum in each cluster.

### Data Collection Procedure

#### Questionnaire Administration

The revised Convergence Insufficiency Symptom Survey questionnaire^20,21^ (Cronbach α of 0.879 for participants) was administered to all selected participants on the day of examination to exclude those with binocular vision anomaly–related symptoms. Asymptomatic participants (score <16)^21^ were selected for the preliminary vision examination.

#### Preliminary Vision Examination

This involved distance and near visual acuity testing using Bailey-Lovie logMAR charts, external examination using handheld slit-lamp biomicroscope, pupillary assessment using a penlight, internal examination using a direct ophthalmoscope (Riester CE-ri-scope L; Riester, Jungingen, Germany), ocular motility using the broad H test, stereoacuity using the TNO stereo test, the Worth four-dot test using a Worth four-dot flashlight, a unilateral cover test with prism neutralization, objective refraction using a static retinoscope (Riester CE ri-scope), and subjective refraction using a trial lens set.

#### Inclusion and Exclusion Criteria

Asymptomatic schoolchildren with unaided visual acuity or maximum plus best-corrected visual acuity of 0.0 or better logMAR were enrolled in the comprehensive binocular vision assessment stage. Participants with ocular diseases, unilateral or bilateral blindness, constant or intermittent strabismus, reduced stereopsis (worse than 60 seconds of arc), suppression, and ocular motility problems were excluded from the comprehensive binocular vision assessment.

#### Comprehensive Binocular Vision Assessment

The outcome parameters measured included near point of convergence using push-up techniques with an accommodative target and a fixation light with red-green anaglyphs, distance and near phoria using the alternate cover test with prism neutralization, near phoria using the modified Thorington test, fusional vergence amplitudes at distance and near using horizontal prism bars, positive relative accommodation, negative relative accommodation, and the gradient accommodative convergence over accommodation ratio. Participants performed each test using their best-corrected spectacle prescription in a trial frame or the phoropter. Only one examiner performed the tests for each of the parameters throughout the study. One examiner performed both near point of convergence techniques, and another examiner performed both phoria techniques. These procedures were followed to avoid interexaminer variability in results. The examiner who performed both near point of convergence test and the other who performed both phoria tests adopted the following masking technique. After the first technique had been performed, the examination form was submitted to an assigned member of the data collection team. This assigned member issued a different examination sheet to the participants and instructed them to join a “second-technique queue” to the same examiner who performed the first technique; the result for this second technique was recorded on the separate examination sheet. The order of testing was randomized to obtain reliable data for the different parameters from active participants.

#### Near Point of Convergence (Push-up Techniques with the Accommodative Target)

The target used was a vertical column of N6 letters on a near-point card. The card was brought from a distance of 50 cm along the facial midline in free space and moved approximately 2 cm/s toward the participants' nose bridge. The card was stopped when the participant reported that the letters were double; no eye turned out to suggest an objective breakpoint. The distance in centimeters from the lateral canthus to the point that the target became double was taken as the breakpoint and recorded. The examiner then pulled the target backward at a speed of approximately 2 cm/s until the participant reported that the letters had become single again. This distance in centimeters from the lateral canthus to this new point (recovery point) was recorded. Measurements were done with a centimeter rule. The procedure was performed twice, and averages were recorded as “break/recovery.”

#### Near Point of Convergence (Fixation Light with Red-green Anaglyphs)

Participants wore red-green anaglyphs, with the red one in front of the right eye and the green one in front of the left eye. A pen torch was positioned at 50 cm along the midline of the participant's face and was brought forward 2 cm/s to the participant's nose bridge. Participants were instructed to report a point at which the red and green color lights were seen separately as two. The distance in centimeters between the lateral canthus and this new point was quickly measured and indicated as the breakpoint. The pen torch was then pulled away from the participant's nose bridge to a point where it became single. This new point (recovery point) was measured in centimeters. The procedure was performed twice, and the averages were recorded as the “break/recovery.”

#### Alternate Cover Test with Prism Neutralization for Distance and Near Heterophoria

Testing was done only in the primary position of gaze as the room lights were kept on so that the participant's eye could be seen with no shadows. The procedure was explained to the participants, and they were instructed to fixate at a target, keeping it clear as they sat upright with their chin and head straight. For distance testing, the target used was a letter on the 6-m Bailey Lovie logMAR chart one line larger in size than the letters on the participant's visual acuity for the poorer eye. For near testing, the target used (single letter, one line better than the participant's near visual acuity of the worse eye on the near card) was positioned at 33 cm and held in line with the participant's visual axis. The occluder was placed before one eye for approximately 2 to 3 seconds and quickly transferred to the other eye. The occluder was kept before the eye for another 2 to 3 seconds as the other eye took up fixation, and then the procedure was repeated. The participants were prevented from viewing the target binocularly at any time. The just-uncovered eye was observed for movements, and any deviation (refixation movements) observed was measured with the prism bar. The amount of prism that neutralized the movement or one prism value below that which reversed the movement was recorded.

#### Modified Thorington Test for Near Heterophoria

The push-button LED lighted Slant Modified Thorington card (Richmond Products Inc., Albuquerque, NM) was used. The red Maddox rod was slotted over the right eye for testing. The red Maddox rod was oriented horizontally and vertically, respectively, in the trial frame for near lateral and vertical phoria testing. The Slant Modified Thorington card was positioned at 40 cm, and participants were instructed to look at the light in the center of the card and to tell the examiner the location of the streak relative to the light. To determine the size of the phoria, the participant was asked to report the target closest to which the streak passed. If the vertical streak passed through the spot of light, orthophoria was recorded through a number (esophoria) and a letter (exophoria). In vertical phoria testing, a right hypophoria and a right hyperphoria were recorded with the corresponding magnitude if the horizontal streak passed through a number and a letter, respectively.

#### Fusional Vergence Amplitudes (Distance and Near)

The prism bar in the free-space (step vergence) method was used to measure fusional vergence amplitudes at distance and near. The target for distance and near measurements was letters one line better than the participant's distance and near visual acuities on 6-m and 40-cm Bailey-Lovie logMAR charts, respectively. The prism bar was oriented base-in and base-out in front of the right eye and increased in magnitude at a speed of approximately 2 seconds for each step to a point where the participants reported that the target became blurry and double (break) and when it became single again (recovery points). For vertical, the breakpoints and recovery points were noted for base-down and base-up at near and distance.

#### Accommodative Convergence Over Accommodation Ratio Determination

Using the gradient method, the cover test heterophoria at near was measured again as +1.00 D lens was added to the participant's near prescription. The accommodative convergence over accommodation ratio was found as the change in heterophoria with +1.00 D lens.

#### Relative Accommodation

The target used for measuring relative accommodation was a line better than the participant's near visual acuity on a near-point card held at 40 cm on a near-point rod in front of the phoropter. The testing was done under bright light. The examiner instructed the participants to keep the target letters single and clear. Plus lenses and minus lenses were increased in 0.25-D intervals before both eyes for negative relative accommodation and positive relative accommodation, respectively, until the participants reported the target was blurred. The final minus lenses and plus lenses were noted for positive relative accommodation and negative relative accommodation, respectively.

### Criteria for the Selection of Participants for the Normative Data Analysis

Participants who did not understand or give clear responses to instructions and/or were unable to report end points for a specific test during the comprehensive binocular vision assessment phase were excluded from the normative data analysis for that parameter. The specific test results for such participants were considered to be unreliable.

### Data Analysis

The IBM SPSS version 21 software was used to analyze the data (IBM SPSS, Armonk, NY). Normative data were described using means ±1 standard deviation with 95% confidence intervals, medians, and maximum and minimum values. An independent-sample *t* test was used to test for significant differences in normative data among demographic parameters. One-way ANOVA with Tukey post hoc test was used to test for significant difference of parameters (in which equal variance was assumed per Levene statistics) among different age groups. Welch ANOVA with Games-Howell post hoc test was used to test for significant difference of parameters (in which equal variances were not assumed per Levene statistics) among different age groups. Pearson correlation coefficient tests were used to test for linear relationships between numerical variables. A one-sample *t* test was used to compare the differences between techniques of specific parameters. Simple linear regression equations were derived to predict specific nonstrabismic binocular vision parameters with age. *P* ≤ .05 was considered statistically significant.

## RESULTS

The Convergence Insufficiency Symptom Survey was administered to 1693 schoolchildren, of which 356 (26.6%) were symptomatic and were excluded from the study. A total of 1337 (73.4%) asymptomatic participants underwent a preliminary vision examination, of whom 76 (5.7%) were excluded because of ocular diseases (1.6%), amblyopia (1.1%), strabismus (0.7%), nystagmus (0.2%), reduced stereoacuity (0.5%), suppression (0.8%), ocular motility problems (0.1%), reading disability (0.1%), photophobia (0.2%), and uncooperative children (0.4%). The remaining 1261 (94.3%) asymptomatic participants (Convergence Insufficiency Symptom Survey score, 7.56 ± 4.99; 95% confidence interval, 7.29 to 7.84) comprising 609 (48.3%) boys and 652 (51.7%) girls with ages ranging from 11 to 17 years (mean, 14.75 ± 1.53 years), 605 (48.0%) of whom were from urban communities and 656 (52%) from rural communities, were chosen to undergo a comprehensive binocular vision assessment. An independent-sample *t* test revealed that the boys were significantly older (14.9 ± 1.6 years) than the girls (14.6 ± 1.4 years; *t*_1259_ = 215; *P* < .001); rural children were significantly older (14.9 ± 1.4 years) than urban children (14.6 ± 1.6 years; *t*_1259_ = −3.194; *P* = .001). The number of participants who experienced difficulty with specific procedures for specific parameters is indicated (Appendix Table A1, available at http://links.lww.com/OPX/A495). The final data reported were normally distributed.

The normative data for the nonstrabismic binocular vision parameters are indicated (Table 1). Accommodative target near point of convergence was statistically significantly lower than fixation light with red-green anaglyph technique (Tables 1, 2); the mean differences in the break of 2.28 cm and recovery of 2.60 cm are clinically meaningful. The mean difference of 0.2Δ between near phoria for the alternate cover test with prism neutralization and the modified Thorington test is not clinically meaningful (Tables 1, 2). All the nonstrabismic binocular vision parameters presented were statistically significantly different among demographic parameters (sex and rural vs. urban) indicated (Table 2); their mean differences, however, were not clinically meaningful.

**TABLE 1:** Expected data for parameters of nonstrabismic binocular vision in Ghanaian children

Parameter investigated	Mean	Standard deviation	95% CI	Minimum value	Maximum value	Median value
AT NPC break	6.10	1.67	6.00 to 6.19	3	10	6
AT NPC recovery	8.17	1.67	8.08 to 8.27	5	12	8
FLRG NPC break	8.51	2.43	8.36 to 8.65	5	15	8
FLRG NPC recovery	10.95	2.60	10.80 to 11.10	7	18	11
CT lateral distance phoria	0.12 Exo	0.79	0.1 to 0.2	Ortho	3 Exo 3 Eso	Ortho
CT lateral near phoria	2.1 Exo	2.3	1.8 to 2.2	Ortho	8 Exo 4 Eso	2 Exo
MTT lateral near phoria	1.9 Exo	2.5	1.8 to 2.0	Ortho	8 Exo 5 Eso	2 Exo
MTT near vertical phoria	Ortho	0.3		Ortho	1 Hyper/hypo	Ortho
BI break distance	10.05	3.65	9.8 to 10.46	4	20	10
BI recovery distance	5.49	3.0	5.31 to 5.67	2	16	4
BO blur distance	11.56	5.34	11.26 to 11.87	4	25	11
BO break distance	18.74	7.1	18.33 to 19.15	6	30	18
BO recovery distance	10.07	5.03	9.77 to 10.36	2	26	10
BD break distance	6.20	2.95	6.03 to 6.37	2	15	6
BD recovery distance	2.94	1.96	2.82 to 3.05	1	12	2
BU break distance	5.37	2.35	5.24 to 5.51	2	12	5
BU recovery distance	2.61	1.76	2.51 to 2.72	1	10	2
BI blur near	12.83	5.19	12.53 to 13.13	4	26	12
BI break near	20.15	6.91	19.75 to 20.55	6	35	20
BI recovery near	12.22	4.98	11.92 to 12.51	4	27	12
BO blur near	15.17	6.47	14.80 to 15.54	4	30	14
BO break near	22.94	7.75	22.50 to 23.39	7	40	24
BO recovery near	13.63	5.67	13.29 to 13.96	3	30	14
BD break near	5.94	3.25	5.75 to 6.13	2	17	5
BD recovery near	2.95	2.50	2.81 to 3.10	1	16	2
BU break near	6.09	3.29	5.90 to 6.28	2	16	5
BU recovery near	3.18	2.79	3.01 to 3.35	1	15	2
AC/A ratio (gradient)	2.80	1.07	2.73 to 2.87	1	7	3
NRA	+2.54	0.75	+2.50 to +2.58	+1.50	+4.00	+2.50
PRA	−2.58	0.81	−2.53 to −2.62	−1.50	−4.00	−2.50

AC/A = accommodative convergence over accommodation; AT = accommodative target; BD = base-down; BI = base-in; BO = base-out; BU = base-up; CI = confidence interval; CT = cover test; Eso = esophoria; Exo = exophoria; FLRG = fixation light with red-green anaglyph; LP = lateral phoria; MTT = modified Thorington test; NPC = near point of convergence; NRA = negative relative accommodation; Ortho = orthophoria; PRA = positive relative accommodation; PU = push-up method; VP = vertical phoria.

**TABLE 2:** Test of differences between techniques for same parameter and between demographic parameters

Parameter	Techniques	*df*	*t*	*P*	Mean difference	95% CI of means difference
NPC break	AT and FLRG	1028	−27.95	.001	−2.28	−2.44 to −2.12
NPC recovery	AT and FLRG	1047	−30.24	.001	−2.60	−2.77 to −2.43
Near LH	Alternate CT PN and MTT	1139	−2.893	.004	−0.2	−0.4 to −0.1
Sex differences in parameters						
Parameters	Male	Female	*t* (*df*)	*P*	Mean difference	95% CI of mean difference
PRA	−2.64 ± 0.82	−2.52 ± 0.79	2.654 (1228)	.008	0.12	0.03 to 0.21
Distance BO blur	11.91 ± 5.52	11.25 ± 5.14	2.009 (1158)	.04	0.66	0.04 to 1.27
Near BO blur	15.90 ± 6.40	14.45 ± 6.46	3.866 (1174)	.001	1.45	0.71 to 2.19
Near BO break	23.94 ± 7.63	21.97 ± 7.76	4.371 (1166)	.001	1.97	1.09 to 2.85
Near BO recovery	14.34 ± 5.69	12.93 ± 5.50	4.13 (1099)	.001	1.41	0.75 to 2.07
Rural and urban differences	Rural	Urban	*t* (*df*)	*P*	Mean difference	95% CI of mean difference
Near LH CT	2.3 Exo ± 2.3	1.9 Exo ± 2.2	3.460 (1213)	.001	0.4	0.2 to 0.7

AT = accommodative target; BO = base-out; CI = confidence interval; CT = cover test; Exo = exophoria; FLRG = fixation light with red-green anaglyph; LH = lateral heterophoria; MTT = modified Thorington test; NPC = near point of convergence; PN = prism neutralization.

Participants were divided into three groups (Table 3) as follows: young teen (ages 11 to 13 years), middle-aged teen (ages 14 and 15 years), and old teen (ages 16 and 17 years). One-way ANOVA and Welch ANOVA test were performed to determine the difference in parameters between groups. Although there were many statistically significant differences between the groups (Table 3), none of these differences were clinically meaningful. Because of the lack of clinically meaningful differences because of age, the result in Table 1 represents the normative data for the overall sample.

**TABLE 3:** Descriptive measures and differences in nonstrabismic binocular vision parameters among age groups

					95% Confidence interval for mean			
Parameter	Age group (y)	Frequency	Mean	Standard deviation	Lower bound	Upper bound	*F* (*df*_1_,*df*_2_)	*P*
AT NPC break	11–13	247	5.87	1.46	5.684	6.049	3.817 (2,1143)	.02
	14–15	522	6.13	1.69	5.983	6.272		
	16–17	377	6.21	1.77	6.027	6.385		
AT NPC recovery	11–13	245	8.06	1.49	7.869	8.245	0.745 (2,1129)	.48
	14–15	523	8.21	1.70	8.062	8.353		
	16–17	364	8.20	1.76	8.019	8.382		
FLRG NPC break	11–13	250	8.90	2.31	8.602	9.178	4.057 (2,1087)	.02
	14–15	496	8.38	2.39	8.166	8.588		
	16–17	344	8.42	2.54	8.152	8.691		
FLRG NPC recovery	11–13	245	11.36	2.43	11.055	11.667	4.071 (2,1126)	.02
	14–15	520	10.85	2.59	10.626	11.072		
	16–17	364	10.81	2.67	10.530	11.080		
MTT near phoria	11–13	245	−1.6	2.5	−1.921	−1.291	4.152 (2,1164)	.02
	14–15	537	−1.8	2.5	−2.043	−1.616		
	16–17	385	−2.2	2.5	−2.422	−1.921		
NRA	11–13	262	2.49	0.76	2.393	2.577	1.068 (2,1228)	.34
	14–15	561	2.57	0.74	2.504	2.627		
	16–17	408	2.53	0.75	2.455	2.602		
PRA	11–13	263	2.45	0.80	2.356	2.550	4.546 (2,1227)	.01
	14–15	558	2.59	0.80	2.527	2.661		
	16–17	409	2.64	0.82	2.563	2.721		

AT = accommodative target; FL = fixation light target; FLRG = fixation light with red-green anaglyph; minus (−) = exophoria; MTT = modified Thorington test; NPC = near point of convergence; NRA = negative relative accommodation; PRA = positive relative accommodation.

There were significant correlations between age (in years) and near point of convergence break using accommodative target (*r*_1146_ = 0.059, *P* = .04), near point of convergence break using fixation light with red-green anaglyph (*r*_1090_ = 0.064, *P* = .04), near point of convergence recovery using fixation light with red-green anaglyph (*r*_1129_ = −0.075, *P* = .01), positive relative accommodation (*r*_1230_ = 0.067, *P* = .02), and the gradient accommodative convergence over accommodation ratio (*r*_903_ = −0.113, *P* = .001). Predicted normal linear regression equations for these parameters were as follows: near point of convergence break using accommodative target = [5.13 + 0.07(age)], near point of convergence break using fixation light with red-green anaglyph = [10.00 − 0.10(age)], near point of convergence recovery using fixation light with red-green anaglyph = [12.83 − 0.13(age)], positive relative accommodation = [2.05 + 0.04(age)], and gradient accommodative convergence over accommodation ratio = [3.97 − 0.08(age)].

## DISCUSSION

In interpreting these normative data presented (Table 1) to guide binocular vision analysis, the main reference descriptive data for comparison are the mean with ±1 standard deviations as presented in other related studies.^1,3,9,11,14,22^ Using this definition, the ranges of normal for the nonstrabismic binocular vision parameters include the following: accommodative target near point of convergence break (4.43 to 7.77 cm) and recovery (6.50 to 9.84 cm), fixation light with red-green anaglyph near point of convergence break (6.80 to 10.94 cm) and recovery (8.35 to 13.55 cm), cover test distance phoria (0.7 esophoria to 0.9 exophoria) and near phoria (0.2 esophoria to 4.4 exophoria), and modified Thorington test near phoria (0.6 esophoria to 4.4 exophoria). The ranges of normative data are +1.79 to +3.29 for negative relative accommodation, −1.77 to −3.39 for positive relative accommodation, and 1.73:1 to 3.87:1 for the accommodative convergence over accommodation ratio.

Some participants who passed the preliminary vision screening recorded higher phoria measurements at near or distance, increasing the magnitude of the respective normative phoria ranges (Table 1). However, applying Sheard's criteria, these participants had enough fusional vergence reserves to compensate for these demands.^3,23^ A moderate to high phoria may not be a problem in the presence of sufficient fusional vergence.

The new linear equations derived serve as a guide for predicting normative data for those nonstrabismic binocular vision parameters using the age of the participant within the study population. The results of data for the equations fall within the normative range presented previously for each specific parameter. The normal gradient accommodative convergence over accommodation ratio of a 14-year-old schoolchild in the central region of Ghana, for example, is predicted to be 3:1 using the equation 3.97 − 0.08(14). The linear relationship between age and accommodative target near point of convergence break is consistent with the findings of a study^24^ conducted among an older Iranian population. In both studies, accommodative target near point of convergence increased by 0.1 cm for each year of age.

The present study investigated normative data for a wider range of parameters of nonstrabismic binocular vision as compared with other studies that were conducted among South African,^11^ Asian,^7,9^ European,^25^ and American^3,26^ participants (Table 4). As demonstrated in Tables 4 and 5, there was no clinically significant difference among the current and previous studies when all parameters were compared. A mean difference greater than the mean standard deviations of the parameters being compared (between the present study and other related ones) is considered clinically meaningful (Tables 4, 5). Comparisons with the only other similar study undertaken on the African continent^11^ did not reveal clinically significant mean differences in near point of convergence break, near negative fusional vergence break and recovery, near positive fusional vergence break and recovery, positive relative accommodation, and negative relative accommodation (Tables 4, 5). Despite the differences in the age ranges of participants in the present study and other related studies conducted in Asia,^7,9^ Europe,^25^ and America^3^ (Table 4), the differences in the parameters are not clinically meaningful (Table 5).

**TABLE 4:** Comparing present study with normative data studies on vergence parameters in South Africa, Asia, Europe, and America

Authors	Present study	Wajuihian^11^	Abraham et al.^7^	Hussaindeen et al.^9^	Costa Lança and Rowe^25^	Scheiman and Wick^3^
Study setting	Ghana	South Africa	India	India (Tamil Nadu)	Portugal	United States
Study population	11 to 17 y	14 to 17 y	10 to 35 y	7 to 18 y	6 to 14 y	
Sample size	>1000	1211	150	936	530	
NPC break	PU 6.10 ± 1.67 FLRG 8.51 ± 2.43	PU 6.88 ± 2.88	FLRG Subj 10–18 y: 7.17 ± 3.16	PU 3 ± 3 FLRG 7 ± 5	PU 6.0 ± 0.3	PU 5 ± 2.5 FLRG 7 ± 4.0
NPC Rec	PU 8.17 ± 1.67 FLRG 10.95 ± 2.60	PU 9.48 ± 3.47	FLRG Subj 10–18 y: 8.63 ± 3.23	PU 4 ± 4 FLRG 10 ± 7	—	PU 7 ± 3 PLRG 10 ± 5.0
Distance LH	CT 0.1 ± 0.8 Exo	—	MTT 10–18 y: 0 ± 1.2	MTT 0.0 ± 1.0 Eso	CT 0.1 ± 0.6 Exo	CT 1.0 ± 2.0 Exo
Near LH	CT 2.1 ± 2.3 Exo MTT 1.9 ± 2.5 Exo	—	MTT 10–18 y: 1.2 ± 2.6 Exo	MTT 0.4 ± 2.0 Exo	CT 1.8 ± 2.6 Exo	CT 3.0 ± 3.0 Exo
Near VH	MTT 0 ± 0.3	—	MTT 10–18 y: 0.1 ± 1.1	MTT 0.0 ± 0.5	—	—
D NFV break	PB 10.05 ± 3.65	—	—	PB 8 ± 2	—	PB
D NFV Rec	PB 5.49 ± 3.00	—	—	PB 6 ± 2	—	PB
D PFV (blur)	PB 11.56 ± 5.34		—	—	—	PB
D PFV break	PB 18.74 ± 7.1	—	—	PB 17 ± 8	—	PB
D PFV Rec	PB 10.07 ± 5.03	—	—	PB 12 ± 7	—	PB
N NFV blur	PB 12.83 ± 5.19	—	—	—	—	—
N NFV break	PB 20.15 ± 6.91	PB 17.37 ± 5.45	—	PB 15 ± 4	9.7 ± 1.9	—
N NFV Rec	PB 12.22 ± 4.98	PB 12.52 ± 4.23	—	PB 11 ± 4	—	—
N PFV blur	PB 15.17 ± 6.47		—	—	—	PB
N PFV break	PB 22.94 ± 7.75	PB 25.38 ± 9.16	—	PB 26 ± 10	20.2 ± 5.0	PB 23 ± 8 (7–12 y)
N PFV Rec	PB 13.63 ± 5.67	PB 17.49 ± 6.77	—	PB 21 ± 10	—	PB 16 ± 6 (7–12 y)
AC/A ratio	Gradient 2.8 ± 1.07		—	Calculated 5.4 ± 0.6		NS 4 ± 2
NRA	+2.54 ± 0.75	+2.17 ± 0.48	—	—		+2.00 ± 0.50
PRA	−2.58 ± 0.81	−2.44 ± 0.71	—	—		−2.37 ± 1.00

AC/A = accommodative convergence over accommodation; AT = accommodative target; CT = cover test; D = distance; dash (—) = not reported in the study because it was not applicable to the study aim; Eso = esophoria; Exo = exophoria; FLRG = fixation light with red and green filters; LH = lateral heterophoria; MTT = modified Thorington test; N = near; NFV = negative fusional vergence; NPC = near point of convergence; NRA = negative relative accommodation; NS = method not specified; PB = prism bar; PFV = positive fusional vergence; PRA = positive relative accommodation; PU = push-up; Rec = recovery; Subj = subjective; VH = vertical heterophoria.

**TABLE 5:** No clinically meaningful differences between the present study and previous studies

	Related study				
	Wajuihian^11^	Hussaindeen et al.^9^	Abraham et al.^7^	Costa Lança and Rowe^25^	Scheiman and Wick^3^
Parameter	Mean difference	Mean difference	Mean difference	Mean difference	Mean difference
Age range,	14–17	7–18	10–18	6–14	
NPC FLRG (break; cm)	—	1.51	1.34	—	1.51
NPC FLRG (recovery; cm)	—	0.25	2.32	—	0.95
Distance LH	—	—	—	CT 0.02Δ	CT 0.88Δ
Near LH	—	MTT 1.5Δ	MTT 0.7Δ	CT 0.3Δ	CT 0.9Δ
Near VH	—	MTT 0	MTT 0.12Δ	—	—
NFV distance (break)	—	PB 2.05Δ	—	—	—
NFV distance (recovery)	—	PB 0.51Δ	—	—	—
PFV distance (break)	—	PB 1.74Δ	—	—	—
PFV distance (recovery)	—	PB 1.93Δ	—	—	—
NFV near (break)	PB 2.78Δ	PB 5.15Δ	—	—	—
NFV near (recovery)	PB 0.30Δ	PB 1.22Δ	—	—	—
PFV near (break)	2.44Δ	PB 3.06Δ	—	—	—
PFV near (recovery)	3.86Δ	PB 7.37	—	—	—
NRA	0.37D	—	—	—	0.54D
PRA	0.14D	—	—		0.21D

CT = cover test; FLRG = fixation light with red-green anaglyph; LH = lateral heterophoria; MTT = modified Thorington test; NFV = negative fusional vergence; NRA = negative relative accommodation; PB = prism bar; PFV = positive fusional vergence; PRA = positive relative accommodation.

Scheiman et al.^18^ considered a difference in near point of convergence of more than 2 cm to be clinically meaningful. Also, considering age-related mean near point of convergence of approximately 8 and 10 cm in 20- to 30- and 40- to 49-year-old normal participants in a normative data study among an Iranian population,^24^ the difference of approximately 2 cm is very clinically meaningful, considering the many different years in the age range. The two techniques, namely, accommodative target and fixation light with red-green anaglyph near point of convergence in the present study, therefore cannot be used interchangeably among the study population; prior studies^18,26,27^ have reported similar findings for near point of convergence using accommodative and red-green targets in normal subjects. The accommodative target engages different aspects of convergence, namely, accommodative, proximal, and fusional, and thus produces accurate results compared with other targets.^28,29^ The fixation light targets are known to produce more variability in measurement compared with the accommodative target and thus are less recommended.^30^ Three studies,^18,31,32^ however, recommend the use of both techniques on a single subject, to help diagnose convergence insufficiency.

The technique used to measure the near point of convergence in the present study differed from that described in the previous studies,^9,11,18^ which were designed to develop normative values. This makes a direct comparison to previous normative data problematic, highlighting a study limitation. The measurement was performed in free space with the use of measurement rods. There is, however, no consensus on the best zero reference point for the near point of convergence, as studies^7,9,11,18^ have used varying points (nose bridge, spectacle plane, temporal canthus). The use of the temporal canthus in the present study is, in the opinion of the authors, justifiable because the near point of convergence lies in the plane of the center of rotation of the eyes,^23^ which are closer to the temporal canthi region.^33^ This distance is expected to be longer than measures taken from other reference points.^33^

As a normative data study, the focus was to identify participants with normal binocular vision status to investigate these parameters. It was, however, difficult to identify participants with asymptomatic binocular vision problems, as the use of other existing population-based normative values to diagnose anomalies (based on the signs) would have introduced a bias. It is possible that, with the administration of the Convergence Insufficiency Symptom Survey, asymptomatic subjects with binocular vision problems may have been included in the study. To help control this, however, it was ensured that all asymptomatic participants (mean Convergence Insufficiency Symptom Survey score, 7.56 ± 4.99) were only included in the comprehensive binocular vision assessment if they had normal stereopsis, normal ocular motility, no ocular suppression, and no strabismus. These measures (stereopsis, suppression, and ocular motility) are important determinants of a normal single binocular vision system.^23^

Because most of the techniques were done in free space, the authors acknowledge as a limitation the difficulty to maintain a steady target. Furthermore, it is acknowledged that, during the training session for examiners, the reliability and validity of specific test measurements were observational and not analyzed through statistical methods. Moreover, it is difficult to determine the extent to which these population-expected data may be applied to a single patient; this is seen to be the major limitation with normative data studies.^22,34^ Notwithstanding, the normative data serve as a guide for evaluating and managing nonstrabismic binocular vision problems among junior high school children in Ghana. The data presented are delimited to the tests and techniques used and must be analyzed from the perspective of the limitations indicated.

## Supplementary Material

SUPPLEMENTARY MATERIAL

## References

[1] Morgan MW. Analysis of Clinical Data. Am J Optom Arch Am Acad Optom 1944;21:477–91.

[2] American Optometric Association (AOA). Care of the Patient with Accommodative and Vergence Dysfunction. Available at: https://www.aoa.org/AOA/Documents/Practice%20Management/Clinical%20Guidelines/Consensus-based%20guidelines/Care%20of%20Patient%20with%20Accommodative%20and%20Vergence%20Dysfunction.pdf. Accessed April 16, 2021.

[3] Scheiman M Wick B. Clinical Management of Binocular Vision: Heterophoric, Accommodative and Eye Movement Disorders. 4th ed. Philadelphia, PA: Lippincott Williams & Wilkins; 2014.

[4] Lesser SK. Introduction to Modern Analytical Optometry. Duncan, OK: Optometric Extension Program Foundation, Inc.; 1974.

[5] Sheedy JE Saladin JJ. Association of Symptoms with Measures of Oculomotor Deficiencies. Am J Optom Physiol Opt 1978;55:670–6. 747192 10.1097/00006324-197810000-00002

[6] Hussaindeen JR George R Swaminathian M, . Binocular Vision Anomalies and Normative Data (Band) in Tamilnadu—Study Design and Methods. Vis Dev Rehabil 2015;1:260–70.

[7] Abraham NG Srinivasan K Thomas J. Normative Data for Near Point of Convergence, Accommodation, and Phoria. Oman J Ophthalmol 2015;8:14–8. 25709268 10.4103/0974-620X.149856 PMC4333536

[8] Chen AH Abidin AH. Vergence and Accommodation System in Malay Primary School Children. Malays J Med Sci 2002;9:9–15. 22969312 PMC3436106

[9] Hussaindeen JR Rakshit A Singh NK, . Binocular Vision Anomalies and Normative Data (BAND) in Tamil Nadu: Report 1. Clin Exp Optom 2017;100:278–84. 27796049 10.1111/cxo.12475

[10] Majumber C. Comparison of Amplitudes of Accommodation in Different Vertical Viewing Angles. Optom Vis Perform 2015;3:276–80.

[11] Wajuihian SO. Normative Values for Clinical Measures Used to Classify Accommodative and Vergence Anomalies in a Sample of High School Children in South Africa. J Optom 2019;12:143–60. 29887298 10.1016/j.optom.2018.03.005 PMC6612036

[12] Kleinstein RN Jones LA Hullett S, . Refractive Error and Ethnicity in Children. Arch Ophthalmol 2003;121:1141–7. 12912692 10.1001/archopht.121.8.1141

[13] Dadeya S Kamlesh Shibal F. The Effect of Anisometropia on Binocular Visual Function. Indian J Ophthalmol 2001;49:261–3. 12930119

[14] Jimenez R Perez MA Garcia JA, . Statistical Normal Values of Visual Parameters That Characterize Binocular Function in Children. Ophthalmic Physiol Opt 2004;24:528–42. 15491481 10.1111/j.1475-1313.2004.00234.x

[15] Chen AH Iqbal R. The Effect of Refractive Error and Race on the Vergence and Accommodation Systems. J Behav Optom 2000;8:5–8.

[16] Blake CR Lai WW Edward DP. Racial and Ethnic Differences in Ocular Anatomy. Int Ophthalmol Clin 2003;43:9–25. 10.1097/00004397-200343040-00004 14574198

[17] Wang D Huang G He M, . Comparison of Anterior Ocular Segment Biometry Features and Related Factors among American Caucasians, American Chinese and Mainland Chinese. Clin Exp Ophthalmol 2012;40:542–9. 22172206 10.1111/j.1442-9071.2011.02746.x

[18] Scheiman M Gallaway M Frantz KA, . Nearpoint of Convergence: Test Procedure, Target Selection, and Normative Data. Optom Vis Sci 2003;80:214–25. 12637833 10.1097/00006324-200303000-00011

[19] Jiménez R González MD Pérez MA, . Evolution of Accommodative Function and Development of Ocular Movements in Children. Ophthalmic Physiol Opt 2003;23:97–107. 12641697 10.1046/j.1475-1313.2003.00093.x

[20] Borsting E Rouse MW Deland PN, . Association of Symptoms and Convergence and Accommodative Insufficiency in School-age Children. Optometry 2003;74:25–34. 12539890

[21] Borsting EJ Rouse MW Mitchell GL, . Validity and Reliability of the Revised Convergence Insufficiency Symptom Survey in Children Aged 9 to 18 Years. Optom Vis Sci 2003;80:832–8. 14688547 10.1097/00006324-200312000-00014

[22] Scheiman M Herzberg H Frantz K, . A Normative Study of Step Vergence in Elementary Schoolchildren. J Am Optom Assoc 1989;60:276–80. 2723323

[23] von Noorden GK Campos EC. Binocular Vision and Ocular Motility: Theory and Management of Strabismus. 6th ed. St. Louis, MO: Mosby; 2002.

[24] Ostadimoghaddam H Hashemi H Nabovati P, . The Distribution of Near Point of Convergence and Its Association with Age, Gender and Refractive Error: A Population Based Study. Clin Exp Optom 2017;100:255–9. 27652584 10.1111/cxo.12471

[25] Costa Lança C Rowe FJ. Variability of Fusion Vergence Measurements in Heterophoria. Strabismus 2016;24:63–9. 27128321 10.3109/09273972.2016.1159234

[26] Maples WC Hoenes R. Near Point of Convergence Norms Measured in Elementary School Children. Optom Vis Sci 2007;84:224–8. 17435536 10.1097/OPX.0b013e3180339f44

[27] Phillips J Tierney R. Effect of Target Type on Near Point of Convergence in a Healthy, Active, Young Adult Population. J Eye Mov Res 2015;8:1–6.

[28] Adler PM Cregg M Viollier AJ, . Influence of Target Type and RAF Rule on the Measurement of Near Point of Convergence. Ophthalmic Physiol Opt 2007;27:22–30. 17239187 10.1111/j.1475-1313.2006.00418.x

[29] Siderov J Chiu SC Waugh SJ. Differences in the Nearpoint of Convergence with Target Type. Ophthalmic Physiol Opt 2001;21:356–60. 11563422 10.1046/j.1475-1313.2001.00609.x

[30] Ciuffreda KJ. Near Point of Convergence as a Function of Target Accommodative Demand. Opt J Rev Optom 1974;111:9–10.

[31] Pang Y Gabriel H Frantz KA, . A Prospective Study of Different Test Targets for the Near Point of Convergence. Ophthalmic Physiol Opt 2010;30:298–303. 20444137 10.1111/j.1475-1313.2010.00731.x

[32] Capobianco NM. The Subjective Measurement of the Near Point of Convergence and Its Significance in the Diagnosis of Convergence Insufficiency. Am Orthopt J 1952;2:40–2. 12985823

[33] Hamed MM David AG Marzieh E. The Relationship between Binocular Vision Symptoms and Near Point of Convergence. Indian J Ophthalmol 2013;61:325–8. 23552348 10.4103/0301-4738.97553 PMC3759101

[34] Shepard CF. The Most Probable “Expected”. Optom Wkly 1941;32:538–41.

