# Supplementary material for: Normative Data for Nonstrabismic Binocular Vision Parameters in African Schoolchildren
Source: Optom Vis Sci. 2021 Jun 3;98(6):620–8. doi: 10.1097/OPX.0000000000001706 (PMC8216600; doi:10.1097/OPX.0000000000001706)
Supplement: SUPPLEMENTARY MATERIAL [file ovs-98-620-s001.docx]

**ORIGINAL INVESTIGATION**

**Normative Data for Non-strabismic Binocular Vision Parameters in African Schoolchildren**

Charles Darko-Takyi, PhD, OD, Vanessa R. Moodley, PhD, MOptom, and Samuel B. Boadi-Kusi, PhD, OD

Discipline of Optometry, University of KwaZulu Natal, South Africa (CD-T, VRM), and Department of Optometry and Vision Science, University of Cape Coast, Ghana (CD-T, SBB-K)

MDT short title: Normative Binocular Vision Data: African Children

5 tables; no figures; 1 appendix

Submitted: February 20, 2020; accepted March 6, 2021.

Corresponding author:

Charles Darko-Takyi

e-mail: cdarko-takyi@ucc.edu.gh/charles.darko-takyi@ucc.edu.gh

**ABSTRACT**

**Significance.** With reported population differences in parameters of non-strabismic binocular vision, the present study investigated and reports normative data among a sample of African children. **Purpose.** To determine expected binocular visual function data among school children in the Central Region of Ghana. **Methods.** The study used a prospective cross-sectional design and employed a multi-stage cluster sampling approach. Eligible normal participants selected through the administration of convergence insufficiency symptom survey (CISS) questionnaire (score ˂ 16) and preliminary vision screening, underwent comprehensive binocular vision testing. Only data for participants who expressed no difficulty with the specific procedures were analyzed. **Results.** A total of 1, 261 normal participants [11- 17 years (mean 14.75 ±1.530 years)] were selected for comprehensive binocular vision testing in the normative data study. The mean with plus-or-minus-one standard deviations for normative data for the non-strabismic binocular vision parameters include: accommodative target near point of convergence (NPC) break (6.10 cm ± 1.67), NPC recovery (8.17 cm ± 1.67), fixation light with red-green anaglyph (RG NPC) break (8.51 cm ± 2.43), RG NPC recovery (10.95 cm ± 2.60), cover test (CT) distance phoria (0.12 Exo ± 0.79), CT near phoria (2.1 Exo ± 2.3),modified Thorington test (MTT) near phoria (1.9 Exo ± 2.5), negative relative accommodation (+2.54 D ± 0.75), positive relative accommodation (PRA) (− 2.58 D ± 0.81) and AC/A ratio (2.80 ± I.07 : 1). Age (in years) predicted normal linear regression equations for NPC break (5.13 + 0.07 × Age), RG NPC break (10.00 - 0.10 × Age), RG NPC recovery (12.83 - 0.13 × Age), PRA (2.05 + 0.04 × Age), and gradient AC/A ratio (3.97 - 0.08 × Age) serve as guide. **Conclusions.** The study provides expected data that optometrists may use with similar aged Black African populations.

Different normative values for parameters of non-strabismic binocular vision exist in literature and have been used worldwide as guidelines to diagnose and treat binocular vision anomalies. Notable among them are Morgan’s table of expected findings,^1^ a modified Morgan’s table,^2^ Optometric Extension Program table of expected findings,^3,4^ Saladin and Sheedy normative values,^5^ and Scheiman and Wick’s table of expected values for binocular vision testing , which is generally considered as the clinical standard.^3^ Reference guidelines for these values date back to old studies mainly conducted on Caucasian American populations,^6^ making the application of these normative values when analyzing binocular vision case results in other ethnic populations challenging.^6,7,8^ Clinicians in the African continent conventionally made the diagnosis and management of non-strabismic binocular vision disorders by comparing their clinic test results with these established standard normative values.^6,7,8^ This practice, however, may be inappropriate as literature acknowledges population differences in normative data for visual function parameters of non-strabismic binocular vision.^9,10,11^ Variables such as race, ethnicity, and age define a population and are known to influence refractive status^7,12^ and in turn binocular vision,^13,14,15^ due to the differences in ocular anatomy.^16,17^ There is, therefore, a need for population-specific normative data for parameters of non-strabismic binocular vision.

All known binocular vision case analysis techniques require clinic test results to be compared to established normative data.^3^ This is a pre-requisite to classifying patients as having normal or abnormal binocular vision status.^6,9^ Recently, studies have sought to define cut off normative clinical values for specific tests and thus introduced different values among different populations.^7,9,11,14,18,19^ According to Hussaindeen et al.,^9^ “to optimize the sensitivity and specificity of diagnosis, ethnicity-specific cut-off values for binocular vision parameters are mandatory”.

In Africa, there is a single study among South African children^11^ which reported normative data for visual function parameters for non-strabismic binocular vision. If one considers the evidence of ethnic variability, these data are technically applicable to 14 to 17-year-old Black South African children only. In the absence of additional African studies to determine normative data, the applicability of the parameters found to other African children remains unknown. Access to education is growing in Africa and children are exposed to an exponential increase in near point devices with their associated demands on the binocular vision system. To ensure accurate and reliable diagnosis and management of non-strabismic binocular vision disorders in African children, there is the need for standard population-specific normative data for the different parameters of non-strabismic binocular vision disorders. This study was designed to sample normal junior high school children in the central region of Ghana to determine normative data for visual function parameters of non-strabismic binocular vision. It will also provide an opportunity to compare, not only with the Caucasian population studies but also with the non-strabismic binocular vision findings of another African sample.

**METHODS**

**Ethical Consideration**

This population-based, prospective, cross-sectional study conformed to the tenets of the Declaration of Helsinki and was ethically approved by the Biomedical Research Ethics Committee of the University of KwaZulu Natal and Ghana Health Service Ethics Review Committee. Parents and guardians of participants gave written informed consent and participants gave written assent after the authors explained the study to them. Each participant was educated that they could opt-out of the study at any stage if they wished to do so.

**Participant Selection**

The study employed a multi-stage, stratified, cluster sampling technique to select participants. The twenty districts within the central region of Ghana were clustered into five based on their proximity. The various localities within the districts in each cluster were stratified into two namely rural and urban areas based on the Ghana 2010 Population and Housing Census definition. Within each of the strata, one or two schools were randomly selected from each cluster. Children in the first selected school were randomly selected and examined and in cases where their numbers were not up to that required for the study, children in the second selected school were recruited into the study.

Estimated normative minimum sample sizes were calculated for each of the non-strabismic binocular vision parameters (see Appendix Table A1, available at http://links.lww.com/OPX/A495) using the formulae **n= [Z_1-α/2_^2^ SD^2^]/d^2^,** where **Z_1-α/2_** represented standard normal variate at 95% confidence interval (P<0.05) = 1.96; **SD** represented **s**tandard deviation of current standards^3^ and **d** represented absolute allowable error in estimating values. As indicated (see Appendix Table A1, available at http://links.lww.com/OPX/A495), the minimum sample size of 984 was ideal to determine normative data for the parameter with the highest standard deviation to achieve acceptable precision. With an expected minimum sample size of 197 for each cluster, several not less than 99 normal participants were expected to be randomly selected from each rural and each urban stratum in each cluster.

**Data Collection Procedure**

***Questionnaire Administration***

The revised convergence insufficiency symptoms survey questionnaire ^20, 21^ (Cronbach alpha of 0.879 for participants) was administered to all selected participants on the day of examination to exclude those with binocular vision anomaly related symptoms. Asymptomatic participants (score ˂ 16)^21^ were selected for the preliminary vision examination.

***Preliminary Vision Examination***

This involved distance and near visual acuity testing using Bailey-Lovie LogMAR charts, external examination using hand-held slit lamp biomicroscope, pupillary assessment using a penlight, internal examination using a direct ophthalmoscope (Riester CE-ri-scope L), ocular motility using broad H test, stereo acuity using TNO stereo test, Worth-four-dot test using a worth-four-dot flashlight, unilateral cover test with prism neutralization, objective refraction using static retinoscope (Riester CE ri-scope) and subjective refraction using a trial lens set.

***Inclusion and Exclusion Criteria***

Asymptomatic school children, with unaided visual acuity or maximum plus best-corrected visual acuity of 0.0 or better logMAR were enrolled in the comprehensive binocular vision assessment stage. Participants with ocular diseases, unilateral or bilateral blindness, constant or intermittent strabismus, reduced stereopsis (worse than 60 sec/arc), suppression, and ocular motility problems were excluded from the comprehensive binocular vision assessment.

***Comprehensive Binocular Vision Assessment***

The outcome parameters measured included near point of convergence using push-up techniques with an accommodative target and a fixation light with red-green anaglyphs, distance and near phoria using the alternate cover test with prism neutralization, near phoria using modified Thorington test, fusional vergence amplitudes at distance and near using horizontal prism bars, positive relative accommodation, negative relative accommodation, and the gradient AC/A ratio. Participants performed each test using their best-corrected spectacle prescription in a trial frame or the phoropter. Only one examiner performed the tests for each of the parameters throughout the study. One examiner performed both near point of convergence techniques and another examiner performed both phoria techniques. These procedures were followed to avoid inter-examiner variability in results. The examiner who performed both near point of convergence test and the other who performed both phoria tests adopted the following masking technique. After the first technique had been performed, the examination form was submitted to an assigned member of the data collection team. This assigned member issued a different examination sheet to the participants and instructed them to join a “second-technique-queue” to the same examiner who performed the first technique; the result for this second technique was recorded on the separate examination sheet. The order of testing was randomized to obtain reliable data for the different parameters from active participants.

***Near Point of Convergence (Push-up Techniques with the Accommodative Target)***

The target used was a vertical column of N6 letters on a near point card. The card was brought from a distance of 50cm along the facial midline in free space and moved approximately 2 cm per second toward the participants’ nose bridge. The card was stopped when the participant reported that the letters were double; no eye turned out to suggest an objective breakpoint. The distance in centimeters from the lateral canthus to the point that the target became double was taken as the breakpoint and recorded. The examiner then pulled the target backward at a speed of approximately 2 cm per second until the participant reported that the letters had become single again. This distance in centimeters from the lateral canthus to this new point (recovery point) was recorded. Measurements were done with a centimeter rule. The procedure was performed twice and averages recorded as “Break / Recovery”.

***Near Point of Convergence (Fixation Light with Red-green Anaglyphs)***

Participants wore red-green anaglyphs with the red one in front of the right eye and the green one in front of the left eye. A pen torch was positioned at 50cm along the midline of the participant’s face and was brought forward 2cm per second to the participant’s nose bridge. Participants were instructed to report a point at which the red and green color lights were seen separately as two. The distance in centimeters between the lateral canthus and this new point was quickly measured and indicated as the breakpoint. The pen torch was then pulled away from the participant’s nose bridge to a point where it became single. This new point (recovery point) was measured in centimeters. The procedure was performed twice and the averages were recorded as the “Break / Recovery”.

***Alternate Cover Test with Prism Neutralization for Distance and Near Heterophoria***

Testing was done only in the primary position of gaze as the room lights were kept on so that the participant’s eye could be seen with no shadows. The procedure was explained to the participant and they were instructed to fixate at a target keeping it clear as they sat upright with their chin and head straight. For distance testing, the target used was a letter on the 6m Bailey Lovie logMAR chart one line larger in size than the letters on participant’s visual acuity for the poorer eye. For near testing, the target used (single letter, one line better than the participant’s near visual acuity of the worse eye on the near card) was positioned at 33cm and held in line with the participant’s visual axis. The occluder was placed before one eye for about two to three seconds and quickly transferred to the other eye. The occluder was kept before the eye for another two to three seconds as the other eye took up fixation, and then the procedure was repeated. The participants were prevented from viewing the target binocularly at any time. The just-uncovered eye was observed for movements and any deviation (re-fixation movements) observed was measured with the prism bar. The amount of prism that neutralized the movement or one prism value below that which reversed the movement was recorded.

***Modified Thorington Test for Near Heterophoria***

The push-button LED lighted Slant Modified Thorington card (Richmond Products Inc) was used. The red Maddox rod was slotted over the right eye for testing. The red Maddox rod was oriented horizontally and vertically respectively in the trial frame for near lateral and vertical phoria testing. The Slant Modified Thorington card was positioned at 40 cm and participants were instructed to look at the light in the center of the card and to tell the examiner the location of the streak relative to the light. To determine the size of the phoria, the participant was asked to report the target closest to which the streak passed. If the vertical streak passed through the spot of light, orthophoria was recorded, through a number (esophoria) and a letter (exophoria). In vertical phoria testing, a right hypophoria and right hyperphoria were recorded with the corresponding magnitude if the horizontal streak passed through a number and a letter respectively.

***Fusional Vergence Amplitudes (Distance and Near)***

The prism bar in the free space (step vergence) method was used to measure fusional vergence amplitudes at distance and near. The target for distance and near measurements was letters one line better than the participant's distance and near visual acuities on 6m and 40cm Bailey-Lovie LogMAR charts respectively. The prism bar was oriented base-in and base-out in front of the right eye and increased in magnitude at a speed of approximately two seconds for each step to a point where the participants reported that the target became blurry, double (break) and when it became single again (recovery points). For vertical, the breakpoints, and recovery points were noted for base-down and base-up at near and distance.

***AC/A Ratio Determination***

Using the gradient method, the cover test heterophoria at near was measured again as +1.00 D lens was added to the participant’s near prescription. The AC/A ratio was found as the change in heterophoria with +1.00 D lens.

***Relative Accommodation***

The target used for measuring relative accommodation was a line better than the participant’s near visual acuity on a near point card held at 40cm on a near point rod in front of the phoropter. The testing was done under bright light. The examiner instructed the participants to keep the target letters single and clear. Plus lenses and minus lenses were increased in 0.25D intervals before both eyes for negative relative accommodation and positive relative accommodation respectively until the participants reported the target was blurred. The final minus lenses and plus lenses were noted for positive relative accommodation and negative relative accommodation respectively.

**Criteria for the Selection of Participants for the Normative Data Analysis**

Participants who did not understand or give clear responses to instructions, and / or were unable to report endpoints for a specific test during the comprehensive binocular vision assessment phase were excluded from the normative data analysis for that parameter. The specific test results for such participants were considered to be unreliable.

**Data Analysis**

The IBM SPSS version 21 software was used to analyze the data. Normative data were described using means ± 1 standard deviation with 95% confidence intervals, medians, maximum and minimum values. An independent sample t-test was used to test for significant differences in normative data among demographic parameters. One-way ANOVA with Tukey posthoc test was used to test for significant difference of parameters (in which equal variance was assumed per Levene’s statistics) among different age groups. Welch ANOVA with Games-Howell post hoc test was used to test for significant difference of parameters (in which equal variances were not assumed per Levene’s statistics) among different age groups. Pearson’s correlation coefficient tests were used to test for linear relationships between numerical variables. A one-sample t-test was used to compare the differences between techniques of specific parameters. Simple linear regression equations were derived to predict specific non-strabismic binocular vision parameters with age. A *P*-value of 0.05 or less was considered statistically significant.

**RESULTS**

The convergence insufficiency symptom survey was administered to 1693 school children, out of which, 356 (26.6%) were symptomatic and were excluded from the study. A total of 1337 (73.4%) asymptomatic participants were taken through preliminary vision examination of which 76 (5.7%) were excluded with ocular diseases (1.6%), amblyopia (1.1%), strabismus (0.7%), nystagmus (0.2%), reduce stereo-acuity (0.5%), suppression ( 0.8%), ocular motility problems (0.1%), reading disability (0.1%), photophobia (0.2%) and uncooperative children (0.4%). The remaining 1,261 (94.3%) asymptomatic participants (CISS score = 7.56 ± 4.99, 95% CI: 7.29 – 7.84) comprising 609 (48.3%) males and 652 (51.7%) females, age range 11 to 17 years (mean 14.75 ± 1.53) with 605 (48.0%) from urban communities and 656 (52%) from rural communities were taken through comprehensive binocular vision assessment. An independent sample t-test revealed that, the males were significantly older (14.9 ± 1.6) than females (14.6 ± 1.4), *t* (1259) = 215; ***P*** ˂ .001; rural children were significantly older (14.9 ± 1.4) than urban children (14.6 ± 1.6), *t* (1259) = − 3.194; ***P*** = .001. The number of participants who experienced difficulty with specific procedures for specific parameters is indicated (see Appendix Table A1, available at http://links.lww.com/OPX/A495). The final data reported were normally distributed.

The normative data for the non-strabismic binocular vision parameters are indicated (Table 1). Accommodative target near point of convergence was statistically significantly lower than fixation light with red-green anaglyph technique (Table 1 and Table 2); the mean difference in the break of 2.28 cm and recovery of 2.60 cm are clinically meaningful. The mean difference of 0.2 ∆ between near phoria for the alternate cover test with prism neutralization and modified Thorington test is not clinically meaningful (Table 1 and Table 2). All the non-strabismic binocular vision parameters presented were statistically significantly different among demographic parameters (gender and rural vs. urban) indicated (Table 2); their mean differences, however, were not clinically meaningful.

Participants were divided into three groups (Table 3) as follows: young teen (ages 11 to 13), mid-age teen (ages 14 and 15) and old teen (ages 16 and 17), and one-way ANOVA and Welch ANOVA test performed to determine the difference in parameters between groups. Although there were many statistically significant differences between the groups (Table 3), none of these differences were clinically meaningful. Because of the lack of clinically meaningful differences due to age, the result in Table 1 represents the normative data for the overall sample.

There were significant correlations between age (in years) and near point of convergence break using accommodative target, *r* (1146) = 0.059, ***P*** = .04, near point of convergence break using fixation light with red-green anaglyph, *r* (1090) = 0.064, ***P*** = .04, near point of convergence recovery using fixation light with red-green anaglyph, *r* (1129) = -0.075, ***P*** = .012, PRA, *r* (1230) = 0.067, ***P*** = .02 and gradient AC/A ratio, *r* (903) = -0.113, ***P*** = .001. Predicted normal linear regression equations for these parameters were as follows: near point of convergence break using accommodative target = [5.13 + 0.07 (Age)], near point of convergence break using fixation light with red-green anaglyph = [10.00 − 0.10 (Age)], near point of convergence recovery using fixation light with red-green anaglyph = [12.83 − 0.13 (Age)], positive relative accommodation = [2.05 + 0.04 (Age)], and gradient AC/A ratio = [3.97 − 0.08 (Age)].

**DISCUSSION**

In interpreting these normative data presented (Table 1) to guide binocular vision analysis, the main reference descriptive data for comparison is the mean with plus-or-minus-one standard deviations (mean ± 1 SD) as presented in other related studies.^1,3, 9,11,14,22^ Using this definition, the range of normal for the non-strabismic binocular vision parameters include: accommodative target near point of convergence break (4.43 – 7.77 cm) and recovery (6.50 – 9.84 cm); fixation light with red-green anaglyph near point of convergence break (6.80 – 10.94 cm) and recovery (8.35 – 13.55 cm); cover test distance phoria (0.7 esophoria to 0.9 exophoria) and near phoria (0.2 esophoria to 4.4 exophoria) and modified Thorington test near phoria (0.6 esophoria to 4.4 exophoria). The range of normative data for negative relative accommodation is +1.79 to +3.29, positive relative accommodation is -1.77 to -3.39 and AC/A ratio is 1.73/1 to 3.87/1.

Some participants who passed the preliminary vision screening recorded higher phoria measurements at near or distance, increasing the magnitude of the respective normative phoria ranges (Table 1). However, applying Sheard’s criteria, these participants had enough fusional vergence reserves to compensate for these demands.^3,23^ A moderate to high phoria may not be a problem in the presence of sufficient fusional vergence.

The new linear equations derived serve as a guide for predicting normative data for those non-strabismic binocular vision parameters using the age of the participant within the study population. The results of data for the equations fall within the normative range presented above for each specific parameter. The normal gradient AC/A ratio of a 14-year-old schoolchild in the central region of Ghana, for example, is predicted to be 3:1 using the equation 3.97 – 0.08 (14). The linear relationship between age and accommodative target near point of convergence break is consistent with the findings of a study^24^ conducted among an older Iranian population. In both studies, accommodative target near point of convergence increased by 0.1 centimeters for each year of age.

The present study investigated normative data for a wider range of parameters of non-strabismic binocular vision as compared to other studies that were conducted among South African^11^, Asian,^7,9^ European^25,^ and American^3,26^ participants (Table 4). As demonstrated in Tables 4 and 5, there was no clinically significant difference amongst the current and previous studies when all parameters were compared. A mean difference greater than the mean standard deviations of the parameters being compared (between the present study and other related ones) is considered clinically meaningful (Table 4 and Table 5). Comparisons with the only other similar study undertaken on the African continent^11^ did not reveal clinically significant mean differences in near point of convergence break, near negative fusional vergence break and recovery, near positive fusional vergence break and recovery, positive relative accommodation, and negative relative accommodation (Table 4 and Table 5). Despite the differences in the age range of participants in the present study and other related studies conducted in Asia^7,9^, Europe^25,^ and America^3^ (Table 4), the differences in the parameters are not clinically meaningful (Table 5).

Scheiman et al., ^18^ considered a difference in near point of convergence of more than 2cm to be clinically meaningful. Also, considering age-related mean near point of convergence of approximately 8cm and 10cm in 20 to 30 and 40 to 49-year-old normal participants in a normative data study among an Iranian population^24^, the differences of approximately 2cm is very clinically meaningful considering the many different years in the age range. The two techniques namely accommodative target and fixation light with red-green anaglyphs near point of convergence in the present study, therefore, cannot be used interchangeably among the study population; prior studies^18,26,27^ have reported similar findings for NPC using accommodative and red-green targets in normal subjects. The accommodative target engages different aspects of convergence namely, accommodative, proximal and fusional, and thus produces accurate results compared to other targets.^28,29^ The fixation light targets are known to produce more variability in measurement compared to the accommodative target and thus are less recommended.^30^ Three studies,^18,31,32^ however, recommend the use of both techniques on a single subject, to help diagnose convergence insufficiency.

The technique used to measure the near point of convergence in the present study differed from that described in the previous studies^9,11,18^ which were designed to develop normative values. This makes a direct comparison to previous normative data problematic, highlighting a study limitation. The measurement was performed in free space with the use of measurement rods. There is, however, no consensus on the best zero reference point for the near point of convergence as studies^7,9,11,18^ have used varying points (nose bridge, spectacle plane, temporal canthus). The use of the temporal canthus in the present study is, in the opinion of the authors, justifiable as the near point of convergence lies in the plane of the center of rotation of the eyes^23^ which are closer to the temporal canthi region.^33^ This distance is expected to be longer than measures taken from other reference points.^33^

As a normative data study, the focus was to identify participants with normal binocular vision status to investigate these parameters. It was, however, difficult to identify participants with asymptomatic binocular vision problems as the use of other existing population-based normative values to diagnose anomalies (based on the signs) would have introduced a bias. It is possible that with the administration of the CISS, asymptomatic subjects with binocular vision problems may have been included in the study. To help control this, however, it was ensured that all asymptomatic participants (mean CISS score, 7.56 ± 4.99) were only included in comprehensive binocular vision assessment if they had normal stereopsis, normal ocular motility, no ocular suppression, and no strabismus. These measures (stereopsis, suppression, and ocular motility) are important determinants of a normal single binocular vision system.^23^

As most of the techniques were done in free space, the authors acknowledge as a limitation, the difficulty to maintain a steady target. Further, it is acknowledged that during the training session for examiners, the reliability and validity of specific test measurements were observational and not analyzed through statistical methods. Moreover, it is difficult to determine the extent to which this population expected data may be applied to a single patient; this is seen to be the major limitation with normative data studies.^22,34^ Notwithstanding, the normative data serve as a guide for evaluating and managing non-strabismic binocular vision problems among junior high school children in Ghana. The data presented is delimited to the tests and techniques used and must be analyzed from the perspective of the limitations indicated.

**ACKNOWLEDGEMENTS**

The authors wish to acknowledge all final year clinical optometry students of the University of Cape Coast, Ghana, who assisted in data collection.

**APPENDIX**

Appendix Table A1, available at http://links.lww.com/OPX/A495. Minimum sample sizes and final sample reported for specific non-strabismic binocular vision parameters. The minimum sample size calculated for each of the parameters is ideal to estimate their normative data to achieve acceptable precision.

**REFERENCES**

1. Morgan MW. Analysis of Clinical Data. Am J Optom Arch Am Acad Optom 1944;21:477-91.

2. American Optometric Association (AOA). Care of the Patient with Accommodative and Vergence Dysfunction. Available at: https://www.aoa.org/AOA/Documents/Practice%20Management/Clinical%20Guidelines/Consensus-based%20guidelines/Care%20of%20Patient%20with%20Accommodative%20and%20Vergence%20Dysfunction.pdf. Accessed April 16, 2021.

3. Scheiman M, Wick B. Clinical Management of Binocular Vision: Heterophoric, Accommodative and Eye Movement Disorders, 4th ed. Philadelphia: Lippincott Williams & Wilkins; 2014.

4. Lesser SK. Introduction to Modern Analytical Optometry. Duncan, OK: Optometric Extension Program Foundation, Inc.; 1974.

5. Sheedy JE, Saladin JJ. Association of Symptoms with Measures of Oculo Motor Deficiencies. Am J Optom Physiol Opt 1978;55:670-6.

6. Hussaindeen JR, George R, Swaminathian M, et al. Binocular Vision Anomalies and Normative Data (Band) In Tamilnadu- Study Design and Methods. Vis Dev Rehabil 2015;1:260-70.

7. Abraham NG, Srinivasan K, Thomas J. Normative Data for Near Point of Convergence, Accommodation, and Phoria. Oman J Ophthalmol 2015;8:14-8.

8. Chen AH, Abidin AH. Vergence and Accommodation System in Malay Primary School Children. Malays J Med Sci 2002;9:9-15.

9. Hussaindeen JR, Rakshit A, Singh NK, et al. Binocular Vision Anomalies and Normative Data (BAND) in Tamil Nadu: Report 1. Clin Exp Optom 2016;100:278-84.

10. Majumber C. Comparison of Amplitudes of Accommodation in Different Vertical Viewing Angles. Optometry and Visual Performance 2015;3:276-80.

11. Wajuihian SO. Normative Values for Clinical Measures Used to Classify Accommodative and Vergence Anomalies in a Sample of High School Children in South Africa. J Optom 2019;12:143-60.

12. Kleinstein RN, Jones LA, Hullett S, et al. Refractive Error and Ethnicity in Children. Arch Ophthalmol 2003;121:1141-7.

13. Dadeya S, Kamlesh, Shibal, F. The Effect of Anisometropia on Binocular Visual Function. Indian J Ophthalmol 2001;49:261-3.

14. Jimenez R, Perez MA, Garcia JA, Gonzalez MD. Statistical Normal Values of Visual Parameters that Characterize Binocular Function in Children. Ophthalmic Physiol Opt 2004;24:528-42.

15. Chen AH, Iqbal R. The Effect of Refractive Error and Race on the Vergence and Accommodation Systems. J. Behav. Optom. 2000;8:5-8.

16. Blake CR, Lai WW, Edward DP. Racial and Ethnic Differences in Ocular Anatomy. Int Ophthalmol Clin 2003;43:9-25.

17. Wang D, Guofu H, Mingguang H, et al. Comparison of Anterior Ocular Segment Biometry Features and Related Factors Among American Caucasians, American Chinese and Mainland Chinese. Clin Experiment Ophthalmol 2012;40:542-9.

18. Scheiman M, Gallaway M, Frantz KA, et al. Near Point of Convergence: Test Procedure, Target Selection, and Normative Data. Optom Vis Sci 2003;80:214-25.

19. Jiménez R, González MD, Pérez MA, García JA. Evolution of Accommodative Function and Development of Ocular Movements in Children. Ophthalmic Physiol Opt 2003;23:97-107.

20. Borsting E, Rouse MW, Deland PN, et al. Association of Symptoms and Convergence and Accommodative Insufficiency in School-Age Children. Optometry 2003;74:25-34.

21. Borsting EJ, Rouse MW, Mitchell GL et al. Validity and Reliability of the Revised Convergence Insufﬁciency Symptom Survey in Children Aged 9 to 18 Years. Optom Vis Sci 2003;80:832-38.

22. Scheiman M, Herzberg H, Frantz K, Margolies M. A Normative Study of Step Vergence in Elementary School Children. J Am Optom Assoc 1989;60:276-80.

23. von Noorden GK, Campos EC. Binocular Vision and Ocular Motility: Theory and Management of Strabismus, 6th ed. St. Louis: Mosby; 2002.

24. Ostadimoghaddam H, Hashemi H, Nabovati P, Yekta A, Khabazkhoob M. The Distribution of Near Point of Convergence and its Association with Age, Gender and Refractive Error: A Population Based Study. Clin Exp Optom 2017;100:255-9.

25. Lanca C, Rowe FJ. Variability of Fusion Vergence Measurements in Heterophoria. Strabismus 2016;24:63-9.

26. Maples WC, Hoenes R. Near Point of Convergence Norms Measured in Elementary School Children. Optom Vis Sci 2007;84:224-8.

27. Phillips J, Tierney R. Effect of Target Type on Near Point of Convergence in a Healthy, Active, Young Adult Population. J Eye Mov Res 2015;8:1-6.

28. Adler PM, Cregg M, Viollier AJ, Woodhouse M. Influence of Target Type and RAF Rule on the Measurement of Near Point of Convergence. Ophthalmic Physiol Opt 2007;27:22-30.

29. Siderov J, Chiu SC, Waugh SJ. Differences in the Near Point of Convergence with Target Type. Ophthalmic Physiol Opt 2001;21:356-60.

30. Ciuffreda KJ. Near Point of Convergence as a Function of Target Accommodative Demand. Opt J Rev Optom 1974;111:9-10.

31. Pang Y, Gabriel H, Frantz KA, Saeed F. A Prospective Study of Different Test Targets for the Near Point of Convergence. Ophthalmic Physiol Opt 2010;30:298-303.

32. Capobianco NM. The Subjective Measurement of the Near Point of Convergence and Its Significance in the Diagnosis of Convergence Insufficiency. Am Orthop J 1952;2:40-2.

33. Hamed M, David AG, Marzieh E. The Relationship between Binocular Vision Symptoms and Near Point of Convergence. Indian J Ophthalmol. 2013; 61:325-8.

34. Shepard CF. The Most Probable ‘‘Expected’’ Optom Wkly 1941;32:538-41.

**Table 1.** Expected data for parameters of non-strabismic binocular vision in Ghanaian children.

| **Parameter investigated** | **Mean** | **Standard Deviation** | **95% CI** | **Minimum value** | **Maximum value** | **Median value** |
| --- | --- | --- | --- | --- | --- | --- |
| AT NPC break | 6.10 | 1.67 | 6.00 — 6.19 | 3 | 10 | 6 |
| AT NPC recovery | 8.17 | 1.67 | 8.08 — 8.27 | 5 | 12 | 8 |
| FLRG NPC break | 8.51 | 2.43 | 8.36 — 8.65 | 5 | 15 | 8 |
| FLRG NPC recovery | 10.95 | 2.60 | 10.80 — 11.10 | 7 | 18 | 11 |
| CT lateral distance phoria | 0.12 Exo | 0.79 | 0.1— 0.2 | Ortho | 3 Exo 3 Eso | Ortho |
| CT lateral near phoria | 2.1 Exo | 2.3 | 1.8 — 2.2 | Ortho | 8 Exo 4 Eso | 2 Exo |
| MTT lateral near phoria | 1.9 Exo | 2.5 | 1.8 — 2.0 | Ortho | 8 Exo 5 Eso | 2 Exo |
| MTT near vertical phoria | Ortho | 0.3 |  | Ortho | 1 hyper/hypo | Ortho |
| BI break distance | 10.05 | 3.65 | 9.8 — 10.46 | 4 | 20 | 10 |
| BI recovery distance | 5.49 | 3.0 | 5.31 — 5.67 | 2 | 16 | 4 |
| BO blur distance | 11.56 | 5.34 | 11.26 — 11.87 | 4 | 25 | 11 |
| BO break distance | 18.74 | 7.1 | 18.33 — 19.15 | 6 | 30 | 18 |
| BO recovery distance | 10.07 | 5.03 | 9.77 — 10.36 | 2 | 26 | 10 |
| BD break distance | 6.20 | 2.95 | 6.03 — 6.37 | 2 | 15 | 6 |
| BD recovery distance | 2.94 | 1.96 | 2.82 — 3.05 | 1 | 12 | 2 |
| BU break distance | 5.37 | 2.35 | 5.24 — 5.51 | 2 | 12 | 5 |
| BU recovery distance | 2.61 | 1.76 | 2.51 — 2.72 | 1 | 10 | 2 |
| BI blur near | 12.83 | 5.19 | 12.53 — 13.13 | 4 | 26 | 12 |
| BI break near | 20.15 | 6.91 | 19.75 — 20.55 | 6 | 35 | 20 |
| BI recovery near | 12.22 | 4.98 | 11.92 — 12.51 | 4 | 27 | 12 |
| BO blur near | 15.17 | 6.47 | 14.80 — 15.54 | 4 | 30 | 14 |
| BO break near | 22.94 | 7.75 | 22.50 — 23.39 | 7 | 40 | 24 |
| BO recovery near | 13.63 | 5.67 | 13.29 — 13.96 | 3 | 30 | 14 |
| BD break near | 5.94 | 3.25 | 5.75 — 6.13 | 2 | 17 | 5 |
| BD recovery near | 2.95 | 2.50 | 2.81 — 3.10 | 1 | 16 | 2 |
| BU break near | 6.09 | 3.29 | 5.90 — 6.28 | 2 | 16 | 5 |
| BU recovery near | 3.18 | 2.79 | 3.01 — 3.35 | 1 | 15 | 2 |
| AC/A ratio (gradient) | 2.80 | 1.07 | 2.73 — 2.87 | 1 | 7 | 3 |
| NRA | +2.54 | 0.75 | +2.50 to +2.58 | +1.50 | +4.00 | +2.50 |
| PRA | − 2.58 | 0.81 | − 2.53 to −2.62 | − 1.50 | − 4.00 | − 2.50 |

NPC = near point of convergence, PU = push-up method, AT = accommodative target, RG = red-green filter, FL = fixation light target, CT = cover test, MTT = modified thorington test, LP = lateral phoria, VP = vertical phoria, BI = base-in, BO = base-out, BU = base-up, BD = base-down, AC/A = accommodative convergence over accommodation, NRA = negative relative accommodation, PRA = positive relative accommodation, Eso = esophoria, Exo = exophoria, Ortho = orthophoria

**Table 2.** Test of differences between techniques for same parameter and between demographic parameters.

| **Parameter** | **Techniques** | **Df** | **t- value** | **p-value** | **Mean difference** | **95% CI of means difference** |
| --- | --- | --- | --- | --- | --- | --- |
| NPC break | AT and FLRG | 1028 | − 27.95 | .001 | − 2.28 | −2.44 to − 2.12 |
| NPC recovery | AT and FLRG | 1047 | − 30.24 | .001 | − 2.60 | − 2.77 to − 2.43 |
| Near LH | Alternate CT PN and MTT | 1139 | − 2.893 | .004 | − 0.2 | − 0.4 to − 0.1 |
| **Gender differences in parameters** | | | | | | |
| **Parameters** | **Males** | **Females** | **(df) t-value** | **p-value** | **Mean difference** | **95% CI of mean difference** |
| PRA | − 2.64 ± 0.82 | − 2.52 ± 0.79 | (1228) 2.654 | .008 | 0.12 | 0.03 — 0.21 |
| Distance BO blur | 11.91 ± 5.52 | 11.25 ± 5.14 | (1158) 2.009 | .036 | 0.66 | 0.04 — 1.27 |
| Near BO blur | 15.90 ± 6.40 | 14.45 ± 6.46 | (1174) 3.866 | .001 | 1.45 | 0.71 — 2.19 |
| Near BO break, | 23.94 ± 7.63 | 21.97 ± 7.76 | (1166) 4.371 | .001 | 1.97 | 1.09 — 2.85 |
| Near BO recovery | 14.34 ± 5.69 | 12.93 ± 5.50 | (1099) 4.13 | .001 | 1.41 | 0.75 — 2.07 |
| **Rural and urban differences** | | | | | | |
|  | **Rural** | **Urban** | **(df) t-value** | **p-value** | **Mean difference** | **95% CI of mean difference** |
| Near LH CT | 2.3 Exo ± 2.3 | 1.9 Exo ± 2.2 | (1213) 3.460 | .001 | 0.4 | 0.2 — 0.7 |

NPC = near point of convergence, AT = accommodative target, FLRG = fixation light with red-green anaglyph, CTPN = cover test with prism neutralization, MTT = modified Thorington test, LH = lateral heterophoria, BO = base-out

Table 3. Descriptive measures and differences in non-strabismic binocular vision parameters among age groups.

| **Parameter** | **Age group (years)** | | **Frequency** | **Mean** | **Std. Deviation** | **95% Confidence Interval for Mean** | | **F (df1,df2)** | ***P*-value** |
| --- | --- | --- | --- | --- | --- | --- | --- | --- | --- |
|  |  |  |  |  |  | **Lower Bound** | **Upper Bound** |  |  |
| AT NPC break | | 11 to 13 | 247 | 5.87 | 1.46 | 5.684 | 6.049 | 3.817 |  |
|  |  | 14 to 15 | 522 | 6.13 | 1.69 | 5.983 | 6.272 | (2,1143) | .022 |
|  |  | 16 to 17 | 377 | 6.21 | 1.77 | 6.027 | 6.385 |  |  |
| AT NPC recovery | | 11 to 13 | 245 | 8.06 | 1.49 | 7.869 | 8.245 | 0.745 |  |
|  |  | 14 to 15 | 523 | 8.21 | 1.70 | 8.062 | 8.353 | (2,1129) | .475 |
|  |  | 16 to 17 | 364 | 8.20 | 1.76 | 8.019 | 8.382 |  |  |
| FLRG NPC break | | 11 to 13 | 250 | 8.90 | 2.31 | 8.602 | 9.178 | 4.057 |  |
|  |  | 14 to 15 | 496 | 8.38 | 2.39 | 8.166 | 8.588 | (2, 1087) | .02 |
|  |  | 16 to 17 | 344 | 8.42 | 2.54 | 8.152 | 8.691 |  |  |
| FLRG NPC recovery | | 11 to 13 | 245 | 11.36 | 2.43 | 11.055 | 11.667 | 4.071 |  |
|  |  | 14 to 15 | 520 | 10.85 | 2.59 | 10.626 | 11.072 | (2,1126) | .02 |
|  |  | 16 to 17 | 364 | 10.81 | 2.67 | 10.530 | 11.080 |  |  |
| MTT near phoria | | 11 to 13 | 245 | -1.6 | 2.5 | -1.921 | -1.291 | 4.152 |  |
|  |  | 14 to 15 | 537 | -1.8 | 2.5 | -2.043 | -1.616 | (2,1164) | .02 |
|  |  | 16 to 17 | 385 | -2.2 | 2.5 | -2.422 | -1.921 |  |  |
| NRA | | 11 to 13 | 262 | 2.49 | 0.76 | 2.393 | 2.577 | 1.068 |  |
|  |  | 14 to 15 | 561 | 2.57 | 0.74 | 2.504 | 2.627 | (2,1228) | .344 |
|  |  | 16 to 17 | 408 | 2.53 | 0.75 | 2.455 | 2.602 |  |  |
| PRA | | 11 to 13 | 263 | 2.45 | 0.80 | 2.356 | 2.550 | 4.546 |  |
|  |  | 14 to 15 | 558 | 2.59 | 0.80 | 2.527 | 2.661 | (2,1227) | .01 |
|  |  | 16 to 17 | 409 | 2.64 | 0.82 | 2.563 | 2.721 |  |  |

AT = accommodative target, NPC = near point of convergence, RG = red-green filter, FL = fixation light target, NRA = negative relative accommodation, PRA = positive relative accommodation, minus (-) = exophoria

**Table 4.** Comparing present study to normative data studies on vergence parameters in Asia, Europe and America**.**

| **Authors** | **Present study** | **Wajuihian^11^** | **Abraham et al.^7^** | **Hussaindeen et al.^9^** | **Lanca and Rowe^25^** | **Scheiman and Wick^3^** |
| --- | --- | --- | --- | --- | --- | --- |
| Study setting | Ghana | South Africa | India | India (Tamil Nadu) | Portugal | USA |
| Study population | 11 to 17 yrs | 14 to 17 yrs | 10 to 35 yrs | 7 to 18 yrs | 6 to 14 yrs |  |
| Sample size | More than 1000 | 1211 | 150 | 936 | 530 |  |
| NPC break | PU 6.10 ±1.67 FLRG 8.51 ± 2.43 | PU 6.88 ± 2.88 | FLRG Subj 10 to 18 yrs: 7.17 ± 3.16 | PU 3 ± 3 FLRG 7 ± 5 | PU 6.0 ± 0.3 | PU 5 ± 2.5 FLRG 7 ± 4.0 |
| NPC Rec | PU 8.17±1.67 FLRG 10.95 ± 2.60 | PU 9.48 ± 3.47 | FLRG Subj 10 to 18 yrs: 8.63 ± 3.23 | PU 4 ± 4 ; FLRG 10 ± 7 | - | PU 7 ± 3 PLRG 10 ± 5.0 |
| Distance LH | CT 0.1 Exo ± 0.8 | - | MTT 10 to 18 yrs: 0 ± 1.2 | MTT 0.0 Eso ± 1.0 | CT 0.1 Exo ± 0.6 | CT 1.0 Exo ± 2.0 |
| Near LH | CT 2.1 Exo ± 2.3 MTT 1.9 Exo ± 2.5 | - | MTT 10 to 18 yrs: 1.2 Exo ± 2.6 | MTT 0.4 Exo ± 2.0 | CT 1.8 Exo ± 2.6 | CT 3.0 Exo ± 3.0 |
| Near VH | MTT 0 ± 0.3 | - | MTT 10 to 18 yrs: 0.1 ± 1.1 | MTT 0.0 ± 0.5 | - | - |
| D NFV break | PB 10.05 ± 3.65 | - | - | PB 8± 2 | - | PB |
| D NFV Rec | PB 5.49 ± 3.00 | - | - | PB 6 ± 2 | - | PB |
| D PFV (blur) | PB 11.56 ± 5.34 |  | - | - | - | PB |
| D PFV break | PB 18.74±7.1 | - | - | PB 17± 8 | - | PB |
| D PFV Rec | PB 10.07±5.03 | - | - | PB 12± 7 | - | PB |
| N NFV blur | PB 12.83 ± 5.19 | - | - | - | - | - |
| N NFV break | PB 20.15 ± 6.91 | PB 17.37 ± 5.45 | - | PB 15± 4 | 9.7 ± 1.9 | - |
| N NFV Rec | PB 12.22 ± 4.98 | PB 12.52 ± 4.23 | - | PB 11±4 | - | - |
| N PFV blur | PB 15.17±6.47 |  | - | - | - | PB |
| N PFV break | PB 22.94±7.75 | PB 25.38 ± 9.16 | - | PB 26±10 | 20.2 ± 5.0 | PB 23 ± 8 (7-12 yrs) |
| N PFV Rec | PB 13.63±5.67 | PB 17.49 ± 6.77 | - | PB 21±10 | - | PB 16 ± 6 (7-12 yrs) |
| AC/A ratio | Gradient 2.8 ± 1.07 |  | - | Calculated 5.4 ± 0.6 |  | NS 4 ± 2 |
| NRA | +2.54 ± 0.75 | +2.17 ± 0.48 | - | - |  | + 2.00 ± 0.50 |
| PRA | −2.58 ± 0.81 | −2.44 ± 0 .71 | - | - |  | -2.37 ± 1.00 |

NPC = Near point of convergence, PB = prism bar PU = push up AT = accommodative target FL = fixation light MTT = Modified thorington test CT = cover test, MTT = Modified thorington test, RP = rotary prisms NFV = Negative fusional vergence, PFV = Positive fusional vergence, FLRG = Fixation light with red and green filters, LH = lateral heterophoria, VH = vertical heterophoria, D = distance, N = near, Rec = recovery, Eso =esophoria, Exo = exophoria,yrs = years, Subj = subjective, - (hyphen) = Not reported in study as not applicable to study aim

**Table 5.** No clinically meaningful differences between the present study and previous studies.

| **Related study** | **Wajuihian^11^** | **Hussaindeen et al.^9^** | **Abraham et al.^7^** | **Lanca and Rowe^25^** | **Scheiman and Wick^3^** |
| --- | --- | --- | --- | --- | --- |
| Age range | 14 – 17 years | 7 – 18 years | 10 – 18 years | 6 – 14 years |  |
| **Parameter** | **Mean difference** | **Mean difference** | **Mean difference** | **Mean difference** | **Mean difference** |
| NPC FLRG (break) | - | 1.51cm | 1.34cm | - | 1.51cm |
| NPC FLRG (recovery) | - | 0.25cm | 2.32cm | - | 0.95cm |
| Distance LH. | - | - | - | CT 0.02∆ | CT 0.88∆ |
| Near LH | - | MTT 1.5∆ | MTT 0.7∆ | CT 0.3∆ | CT 0.9∆ |
| Near VH | - | MTT 0 | MTT 0.12∆ | - | - |
| NFV Distance (break) | - | PB 2.05∆ | - | - | - |
| NFV Distance (recovery) | - | PB 0.51 ∆ | - | - | - |
| PFV Distance (break) | - | PB 1.74∆ | - | - | - |
| PFV Distance (Recovery) | - | PB 1.93∆ | - | - | - |
| NFV Near (break) | PB 2.78∆ | PB 5.15∆ | - | - | - |
| NFV Near (recovery) | PB 0.30∆ | PB 1.22∆ | - | - | - |
| PFV Near (break) | 2.44∆ | PB 3.06∆ | - | - | - |
| PFV Near (recovery) | 3.86∆ | PB 7.37 | - | - | - |
| NRA | 0.37D | - | - | - | 0.54D |
| PRA | 0.14D | - | - |  | 0.21D |

FLRG- fixation light with red-green anaglyph, CT- cover test, MTT- modified Thorington test, PB- prism bar, NFV- negative fusional vergence, PFV- positive fusional vergence, AC/A - accommodative convergence over accommodation, LH- lateral heterophoria, NRA- negative relative accommodation, PRA- positive relative accommodation
